# Supplementary material for: A Non-spiking Neuron Model With Dynamic Leak to Avoid Instability in Recurrent Networks
Source: Front Comput Neurosci. 2021 May 20;15:656401. doi: 10.3389/fncom.2021.656401 (PMC8173185; doi:10.3389/fncom.2021.656401)
Supplement: Supplementary Figure 1 — Signal similarity between the LSM and H-H model. A comparison between the output responses for LSM (green line is the mean across 50 presentations) and the H-H (derived using backward Euler method, blue line is the mean across 50 presentations), for a given pseudo-random sensory input at 50 Hz for each of six sensors (see Figure 3). The responses of the LSM output were offset by 0.1 activity (a.u.) in order to visualize the coherence between the responses of both neuron models. The cross correlation (with zero lag) was 0.99. [file Data_Sheet_1.DOCX]

Supplementary Material

A non-spiking neuron model with dynamic leak to avoid instability in recurrent networks

Udaya B. Rongala^1*^, Jonas M.D. Enander^1^, Matthias Kohler^2^, Gerald E. Loeb^3^, Henrik Jörntell^1^

# Supplementary Figures


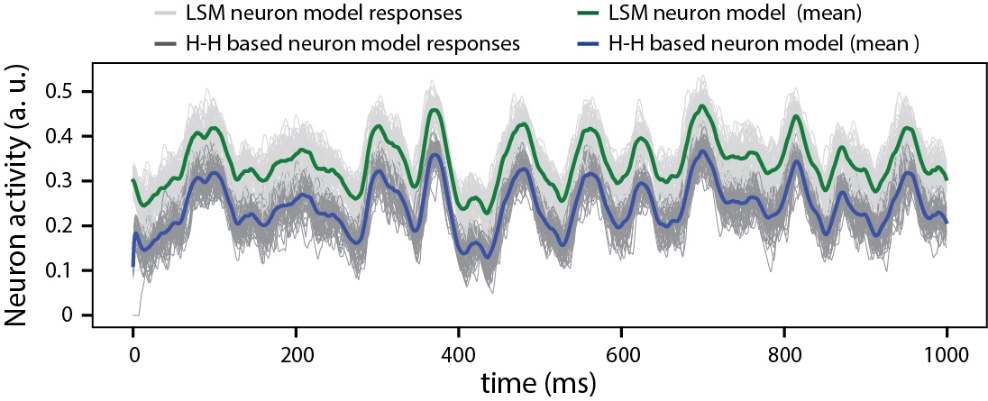


**Supplementary Figure 1. Signal similarity between the LSM and H-H model.** A comparison between the output responses for LSM (green line is the mean across 50 presentations) and the H-H (derived using backward Euler method, blue line is the mean across 50 presentations), for a given pseudo-random sensory input at 50 Hz for each of six sensors (see Figure 3). The responses of the LSM output were offset by 0.1 activity (a.u.) in order to visualize the coherence between the responses of both neuron models. The cross correlation (with zero lag) was 0.99.


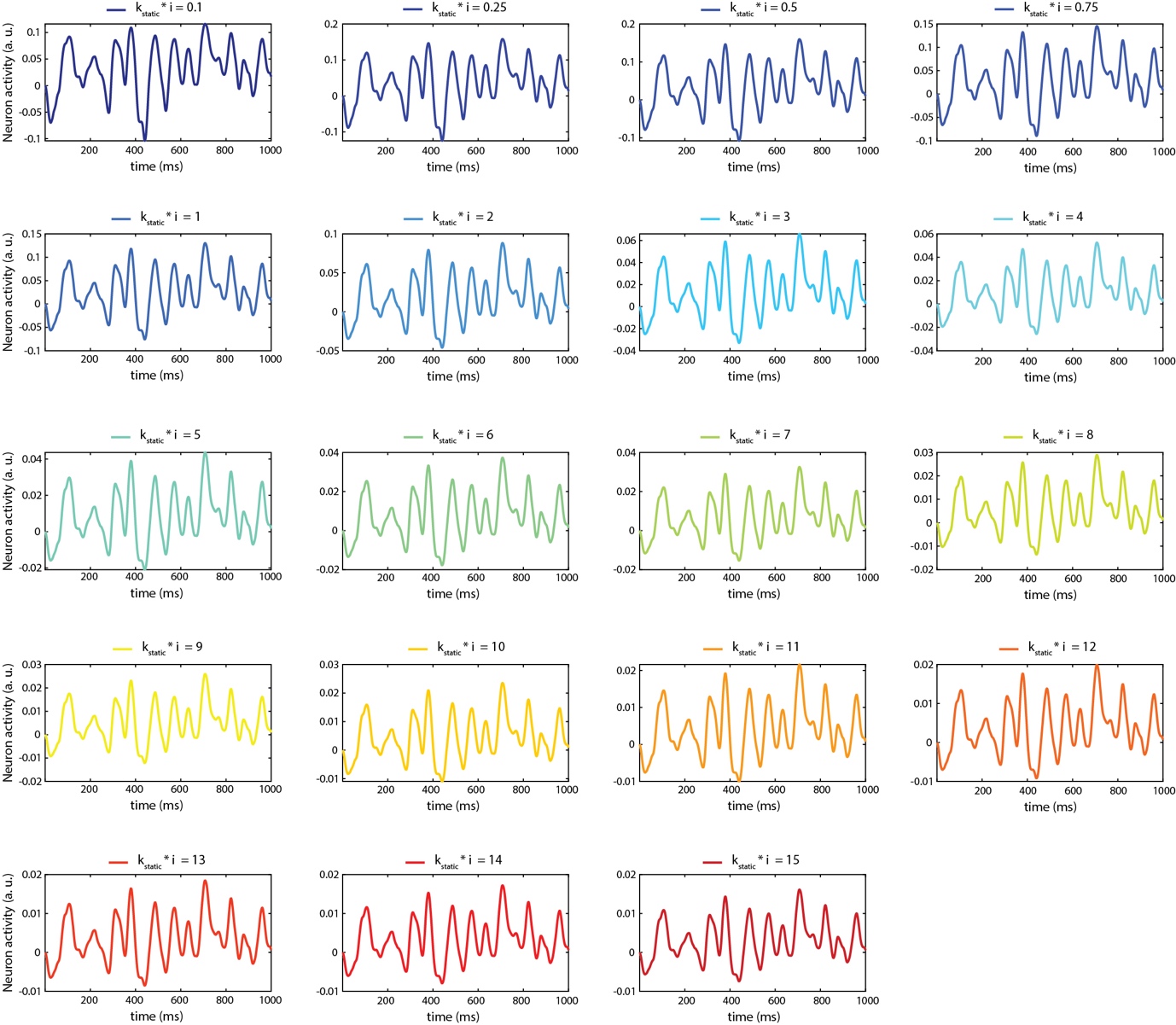


**Supplementary Figure 2.** Impact of the value of $k_{static}$ $k_{\mathrm{static}}$ on the internal activity of the LSM for a given sensory input.


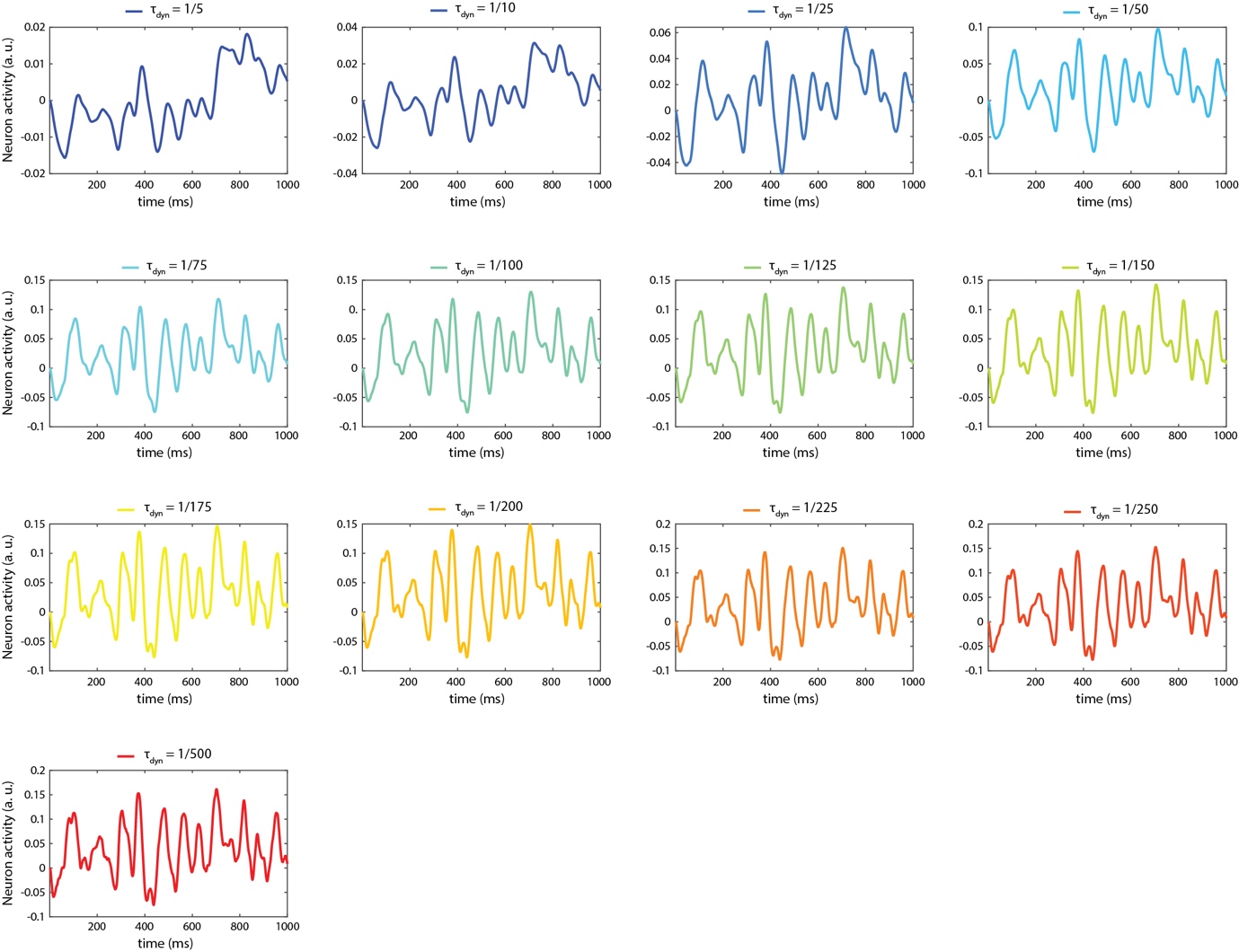


**Supplementary Figure 3.**  Impact of the value of $\tau_{dyn}$ $\tau_{dyn}$ on the internal activity of the LSM for a given sensory input.


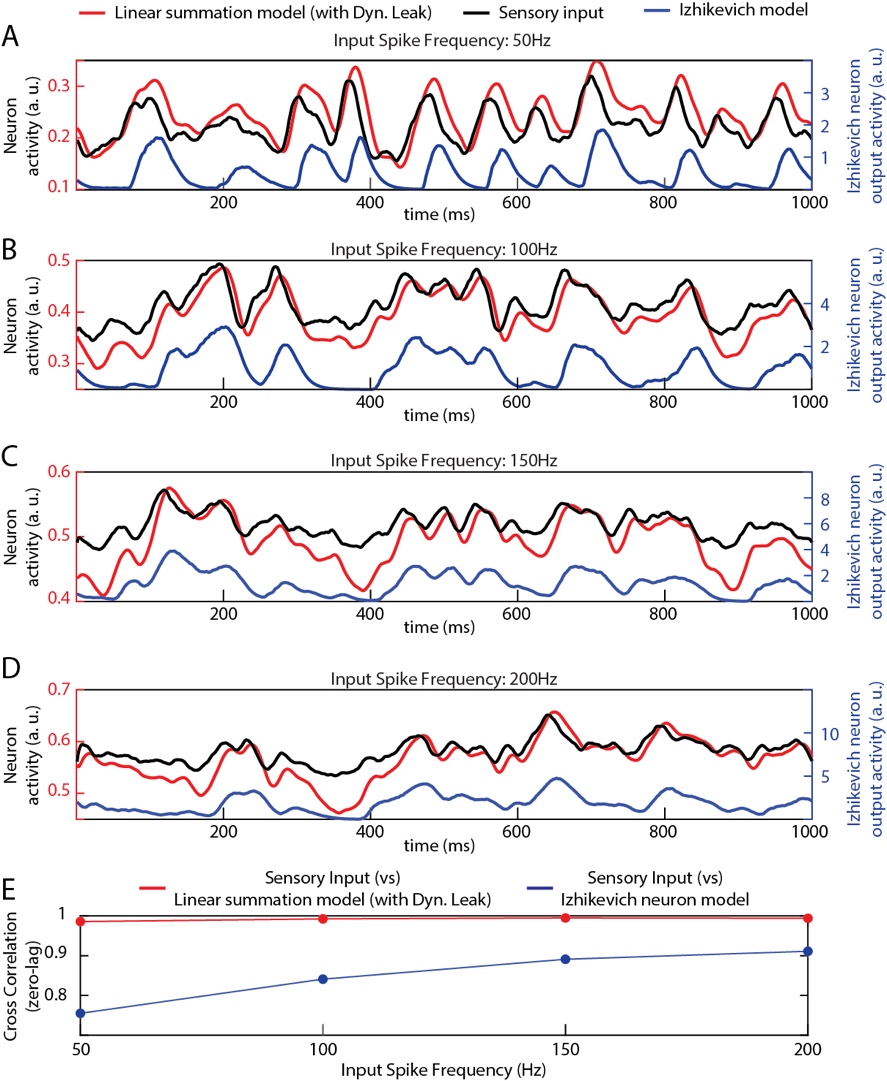


**Supplementary Figure 4. Comparison of the non-spiking and the spiking neuron model outputs for different sensory input frequencies**. (**A-D**) Neuron outputs in response to different sensory input frequencies. (**E**) Cross-correlation between sensory inputs and the neuron model outputs.

**
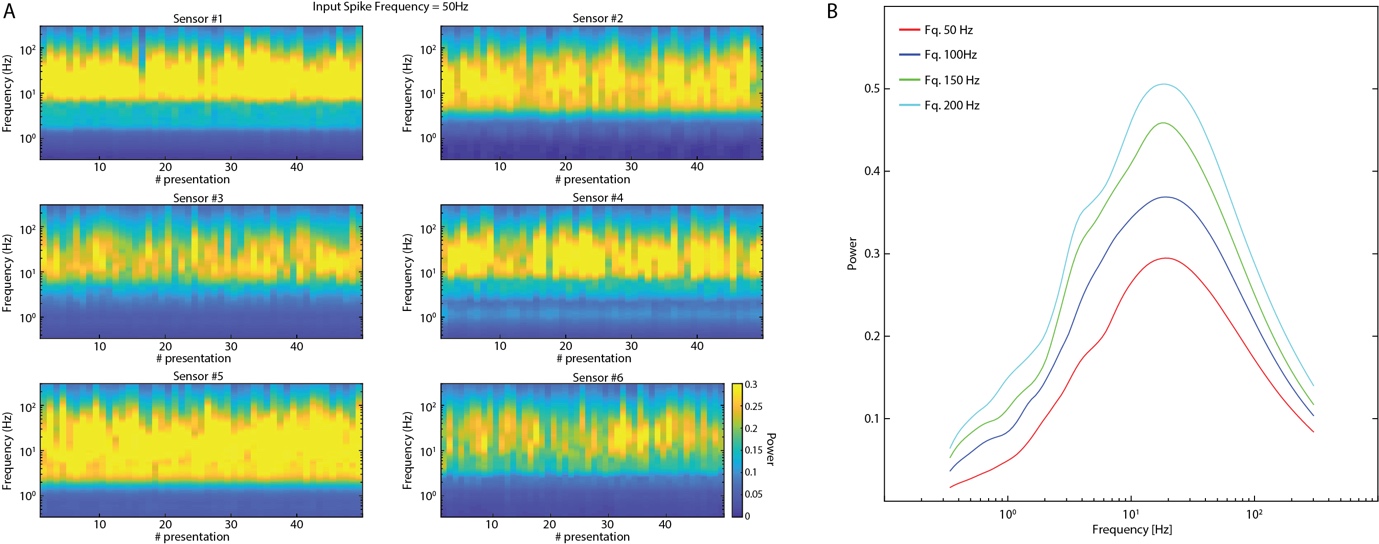
**

**Supplementary Figure 5.** Frequency analysis of the sensory inputs. (**A**) Time-continuous frequency power analysis for each of the six sensory inputs (spike frequency = 50Hz) across the 50 presentations used in the analysis of the network activity. (**B**) Frequency power analysis (using continuous wavelet transform, see Methods), of sensory inputs. The plots show the average power of the activity across all the six sensors, for each of the four mean sensor firing frequencies, across all 50 presentations used in the analysis of the network activity.

**
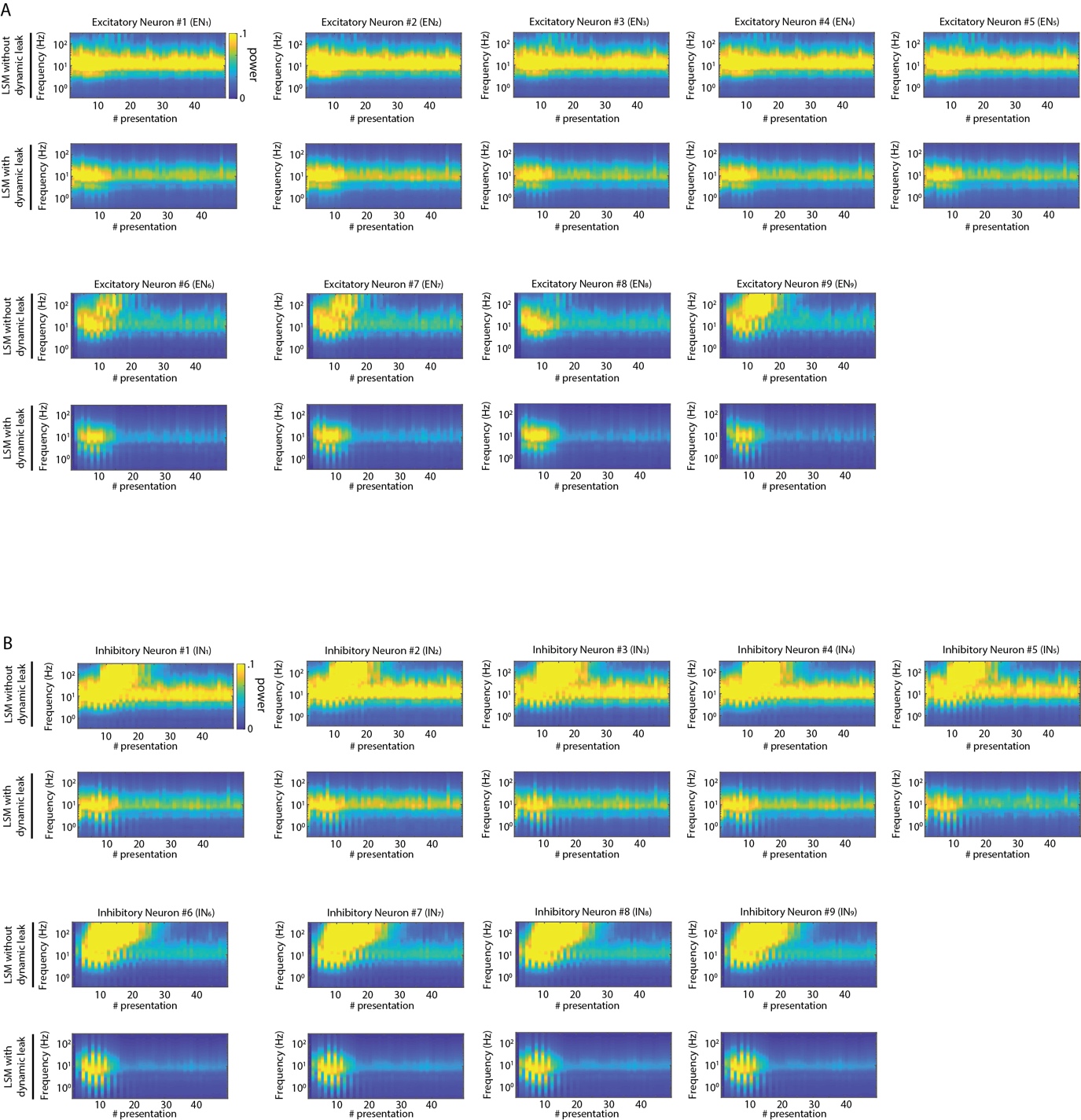
**

**Supplementary Figure 6.** Frequency analysis plots of the activity in all excitatory neurons (${EN}_{1}-{EN}_{9}$) and inhibitory neurons (${IN}_{1}-{IN}_{9}$) for the network shown in Figure 4A.


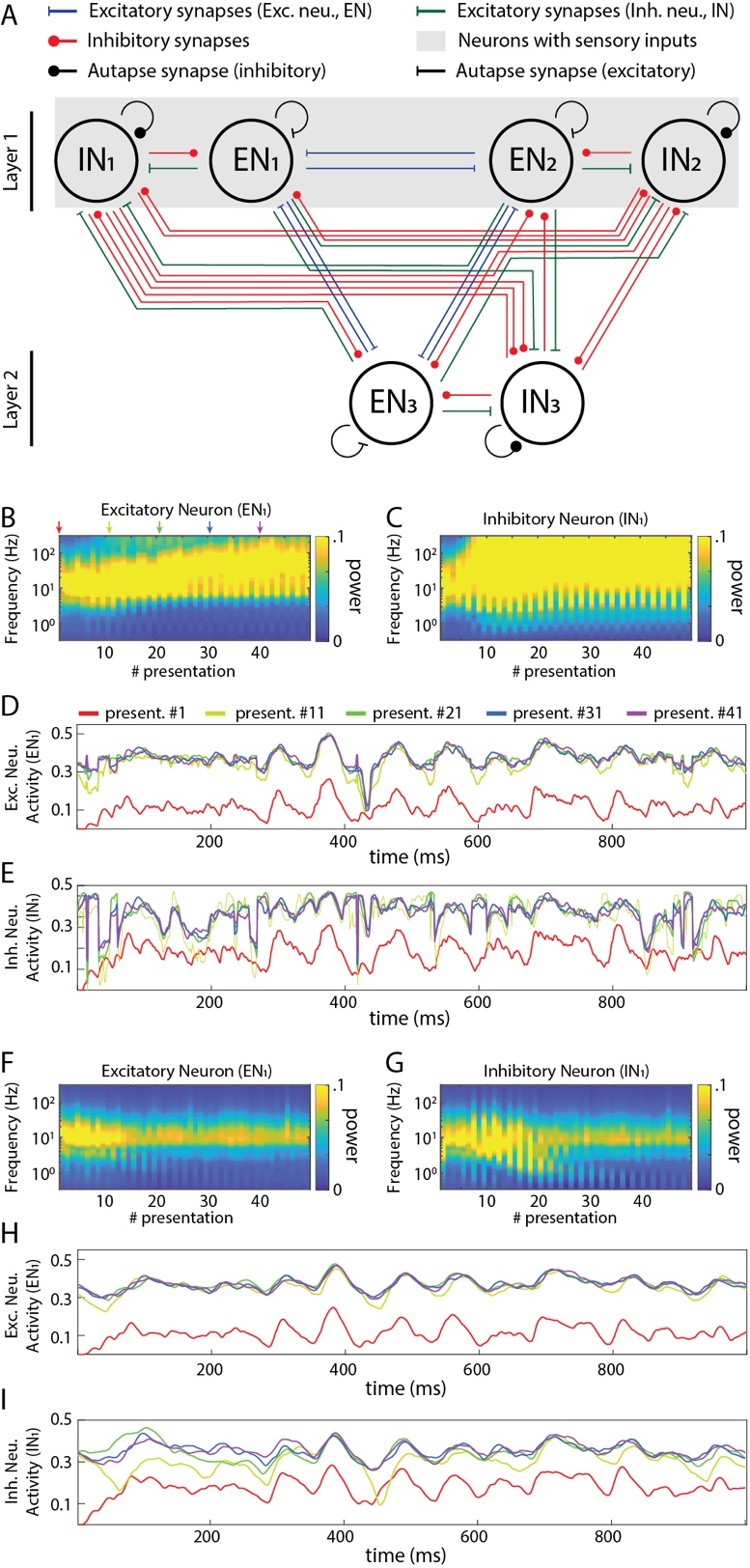


**Supplementary Figure 7. Activity in recurrent networks with autapses**. (**A**) Principles of the connectivity structure in the recurrent network studied. The network presented here is a fully connected network as in Figure 4, with the addition of self-recurrent excitatory and inhibitory synapses (in excitatory and inhibitory neurons, respectively). (**B**) Frequency plot of the activity in an excitatory neuron. (**C**) Similar plot for an inhibitory neuron. (**D**) Raw data plots for sample signals in the excitatory neuron generated at the indicated presentation #. (**E**) Similar plot for the inhibitory neuron. (**F**)-(**I**) Similar plots as in (**B**)-(**E**) but when all the neurons were modelled with the dynamic leak.


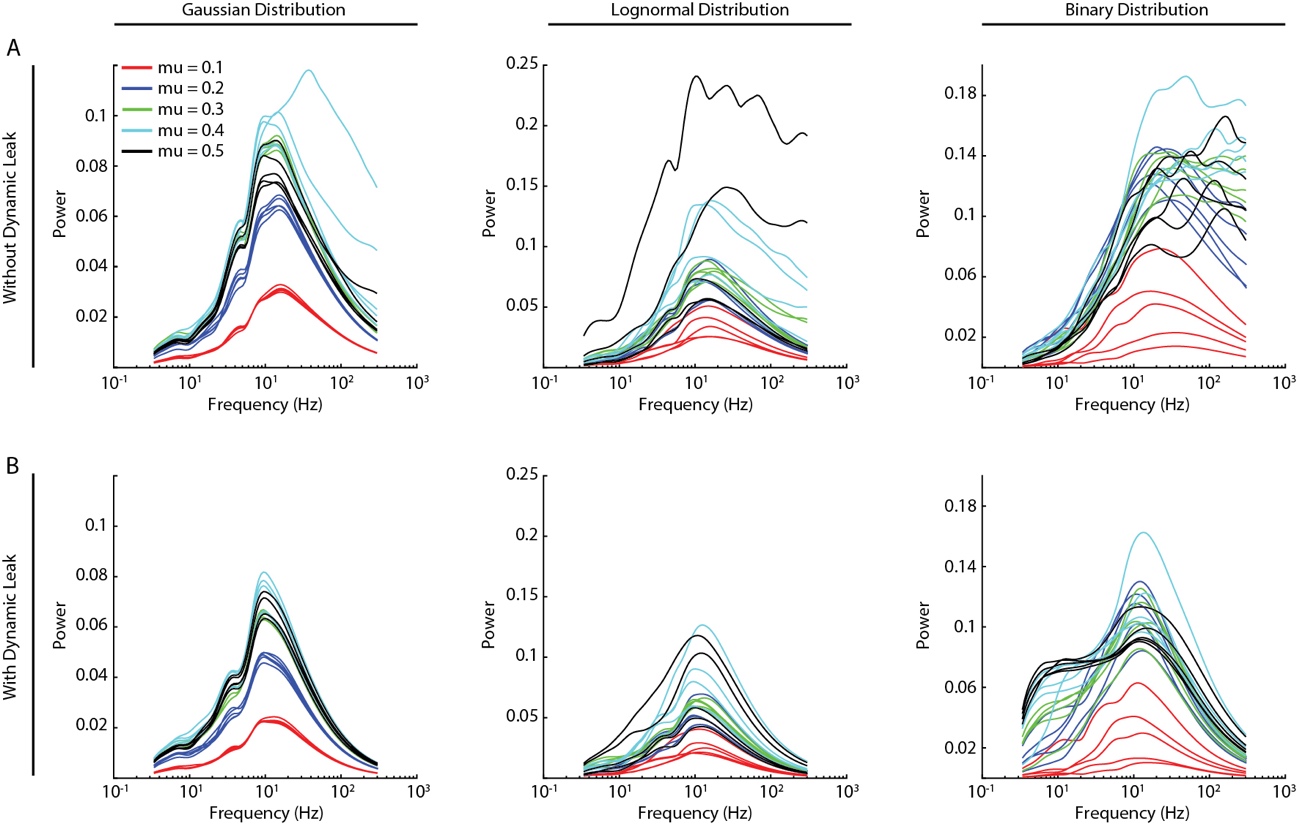


**Supplementary Figure 8. High frequency components and the effect of dynamic leak across different specific synaptic weight distributions**. The synaptic weight distributions used were as shown in Figure 5**A-C**, but instead of representing the 5 random simulations for each setting as an average, we here show them individually. (**A**) The frequency power distributions across all indicated synaptic weight distributions for five randomized repetitions each. Color keys for the different average synaptic weights are the same as in Figure 5 **A-C**. (**B**) Similar display as in **A**, for the same networks but with the neuron model with dynamic leak.

# Supplementary Tables

| **Parameter** | **Symbol** | **Unit** |
| --- | --- | --- |
| Membrane potential | $V$ | [V] |
| Membrane capacitance | $C$ | [F] |
| Membrane time constant | $\tau$ | [s] |
| Time step | $h$ | [s] |
| Neuron output | $v$ | [V] |
| Excitatory weights | $w_{i}$ | [1] |
| Inhibitory weights | $w_{j}$ | [1] |
| Presynaptic excitatory neuron activity | $V_{i}^{+}$ | [V] |
| Presynaptic inhibitory neuron activity | $V_{j}^{-}$ | [V] |
| Excitatory currents | $I_{exc}$ | [A] |
| Inhibitory currents | $I_{inh}$ | [A] |
| Leak currents | $I_{leak}$ | [A] |

**Supplementary Table 1.** H-H Model variable definitions (for the H-H neuron model derivation, presented in Appendix 1).
